# The influence of sense of coherence on psychological birth trauma: a parallel mediation model based on health Salutogenesis theory

**DOI:** 10.3389/fpsyg.2023.1320132

**Published:** 2024-01-05

**Authors:** Tieying Zeng, Lingjun Jiang, Ke Zhang, Meiliyang Wu, Zining Zhu, Zhenjing Hu

**Affiliations:** ^1^Department of Nursing, Tongji Hospital, Tongji Medical College, Huazhong University of Science and Technology, Wuhan, China; ^2^School of Nursing, Tongji Medical College, Huazhong University of Science and Technology, Wuhan, China

**Keywords:** psychological birth trauma, sense of coherence, childbirth-related fear, social support, childbirth readiness, health Salutogenesis

## Abstract

**Background:**

Psychological birth trauma has been associated with a variety of negative consequences for mothers, partners, newborns, and midwives. While prior research has identified demographic and clinical factors that may contribute to the development of psychological birth trauma, interventions targeting these factors can prove challenging. Therefore, the aim of this study was to explore how psychological birth trauma is influenced by psychosocial factors including Sense of Coherence, Childbirth-related Fear, Social Support and Childbirth Readiness.

**Methods:**

A cross-sectional study was conducted in 13 tertiary or secondary hospitals in mainland China from November 2021 to June 2022. One thousand three hundred and sixty-six women were recruited totally. Data regarding basic characteristics, Sense of Coherence (SOC, Sense of Coherence Scale-3), Childbirth-related Fear (CBRF, Fear of Childbirth Scale), Social Support (SS, Medical Outcomes Study Social Support Survey), Childbirth Readiness (CR, Childbirth Readiness Scale) and Psychological Birth Trauma (PBT, Psychological Birth Trauma Scale) were collected. Parallel mediation analysis was adopted to identify the underlying mechanisms between study variables.

**Results:**

It was found that: (1) SOC has been found to have both direct and indirect effects on PBT. Women with higher level of SOC tend to report lower level of PBT; (2) the indirect effect of SOC on PBT was significantly exerted through CBRF, SS and CR; (3) CBRF was found to weaken the protective effect of SOC, whereas SS and CR were found to enhance it. No significant difference was found in contracts of the three specific indirect effects.

**Conclusion:**

SOC, CBRF, SS, and CR should be paid enough attention when designing intervention programs for women who might experience PBT. Interventions targeting SOC and CR are more likely to yield positive outcomes.

## 1 Introduction

Maternal health is a pivotal aspect of public health. With the advancement of society and the improvement of living standards, maternal mental health has garnered unprecedented attention due to its comparable significance to physical health. Psychological birth trauma (PBT), arising from various events during pregnancy or childbirth, is a noteworthy facet of maternal mental health (Taghizadeh et al., 2015). It is characterized by extreme fear, loss of control, helplessness, and powerlessness (Greenfield et al., 2016).

PBT has been extensively studied and its detrimental effects have been confirmed through numerous studies. For women, PBT can result in an unsatisfactory labor experience, increase the likelihood of postpartum depression and post-traumatic stress disorder (PTSD) (James, 2015), and diminish their desire to have more children (Gottvall and Waldenström, 2002; Shorey and Wong, 2022). Furthermore, the adverse effects of childbirth trauma can extend to partners and infants, leading to strained relationships (de Graaff et al., 2018), impaired intimacy (Etheridge and Slade, 2017; Shorey and Wong, 2022), and inadequate feeding for infants (Beck and Watson, 2008). Maternal trauma during childbirth may also trigger traumatic work experiences in midwives, and potentially resulting in PTSD (Beck et al., 2015; Yilmaz Sezer et al., 2023). Evidently, PBT can have far-reaching consequences for all participants in the childbirth event, like ripples in a pond.

A range of factors have been identified as potential causes of PBT. Demographic variables, such as age (Harrison et al., 2021), race (Anderson and Connolly, 2018), economic level (Ghanbari-Homayi et al., 2019), and lack of insurance (Ghanbari-Homayi et al., 2019) have been found to be closely linked to postpartum posttraumatic stress or traumatic birth experience. Clinical factors, including adverse pregnancy history (Nagle et al., 2022), trauma history (Anderson and Connolly, 2018), labor time (Soet et al., 2003; Ghanbari-Homayi et al., 2019), type of delivery (Anderson and Connolly, 2018) pregnancy or birth complications (Ayers et al., 2016) and infant complications (Anderson and Connolly, 2018) have also been shown to be associated with psychological trauma in childbirth. However, these factors are often uncontrollable or only partially controllable, making it difficult to prevent PBT by influencing them. Therefore, many scholars have shifted their focus to highly controllable factors, which may be more amenable to intervention.

To some extent, childbirth should be perceived as a natural and innate process. With the progress of socio-economic development and advancements in medical technology, there is a growing inclination to exert more interventions and control over the childbirth process to ensure more positive maternal and neonatal outcomes. Under the current obstetric service mode, hospital culture may lean toward emphasizing medical interventions to ensure the safety of both mother and child, often overlooking the subjective agency of the parturient herself during the birthing process (Cipolletta, 2016; McKelvin et al., 2021). Many scholars have embarked on an investigation into the psychological reactions of parturients, the forms of service delivery, and the effects of doctor-patient interactions on women’ childbirth experience within the context of an obstetric service mode that prioritizes medical interventions. For women, successful delivery undoubtedly constitutes a positive and gratifying experience. However, the childbirth process is frequently enveloped in the veil of the unknown and uncertainty, yielding a multitude of diverse experiences for parturients. A qualitative study conducted in Italy focusing on primiparous women revealed that despite the universal desire for a smooth childbirth experience, these women were acutely aware of the unpredictability of the birthing process, and manifested concerns and fears regarding this unpredictability (Colciago et al., 2022). Another ethnographic study in Italy also vividly illustrates how both primiparous and multiparous women perceive this sense of uncertainty and fear during the childbirth process (Rania, 2019). Psychological experiences of such fear, validated by studies in Iran and Sweden, have been shown to contribute to negative childbirth experiences among women (Ghanbari-Homaie et al., 2021; Viirman et al., 2022). Research indicates that women often employ intricate psychological mechanisms to cope with the uncertainties and fears associated with childbirth. Their loss of internal control, which was result from their inner fear and uncertain, can lead them to seek external control by relying on healthcare professionals for assistance (Cipolletta and Angela, 2012). This need for a sense of control is also discussed in literature comparing “planned” deliveries (elective cesarean section and vaginal delivery) to “unplanned” deliveries (vacuum extraction or emergency cesarean section) (Handelzalts et al., 2017). These studies, together, emphasized the crucial role of women’s perception of their ability to control uncertain events in shaping their childbirth experiences. Medical interventions, serving as external control resources upon which women depend, also play a pivotal role in the formation of women’s childbirth experiences, as highlighted by various studies in different regions. It was revealed that the provision of comprehensive information and explanations regarding unexpected scenarios or interventions by healthcare providers, coupled with the cultivation of a supportive atmosphere and encouraging doctor-patient relationship, is instrumental in shaping a positive childbirth experience (Dencker et al., 2019; Rania, 2019; McKelvin et al., 2021). Otherwise, unexpected medical interventions during childbirth and inadequate pre-intervention information may contribute to negative childbirth experience, and at times, traumatic birthing experiences (Rania, 2019). These studies collectively highlight the impact of a woman’s attitude toward impending childbirth, her cognitive/psychological preparedness for the birthing process, and the supportive resources she perceived on her ultimate birthing experience. Consequently, it is evident that social and psychological factors indeed play a pivotal role in shaping the childbirth experience of women.

Upon a thorough examination of the literature addressing the impact of social and psychological factors on women’s childbirth experiences, childbirth-related fear (CBRF) emerges as a salient risk factor. Mothers who experience CBRF are more likely to report traumatic birth experience (Ghanbari-Homayi et al., 2019) and develop PTSD (O'Donovan et al., 2014). Social support (SS) has been found to be a protective factor against PBT, particularly for women with a history of previous trauma experience. Both qualitative and quantitative studies have demonstrated that high levels of social support reduce the likelihood of post-traumatic stress symptoms (Soet et al., 2003; McKelvin et al., 2021). Another potential protective factor is Childbirth Readiness (CR), which is reflected by self-management, information literacy, birth confidence, and a birth plan for childbirth (Mengmei et al., 2022). Although there is no direct evidence to show a correlation between CR and PBT, mothers who feel prepared for childbirth were less likely to describe the childbirth process as traumatic (O'Donovan et al., 2014). Meanwhile, adequate maternal information literacy and sufficient pregnancy self-management have been proved to promote physical and psychological preparation for childbirth, strengthen confidence in childbirth, increase the sense of control during the labor process (Akca et al., 2017), and thus reduce the likelihood of PBT. Sense of coherence (SOC), has also been found to be highly correlated with the occurrence of PBT. A meta-synthesis of qualitative studies has linked self-confidence during delivery with positive childbirth experience (Olza et al., 2018). Another meta-analysis combining multiple studies showed that high levels of psychological coherence were associated with low levels of post-traumatic stress symptoms (Schäfer et al., 2019). Therefore, as a psychological trauma, PBT is highly likely to be affected by SOC. These highly controllable factors, namely SOC, CBRF, SS, and CR, have the potential to positively impact the prevention of PBT if properly formulated and adapted.

For too long, healthcare professionals have focused primarily on extrinsic interventions to improve individuals’ health, neglecting the potential of intrinsic factors that can aid in coping with challenges and maintaining health. In the 1980s, Israeli scholar Aaron Antonovsky proposed the theory of health Salutogenesis, which suggests that an individual’s SOC can enhance their ability to select general resistance resources (internal and external) from their environment that may benefit their health (Antonovsky, 2022). Internal resources include past experiences, cognitive abilities, problem-solving skills, and others, while external resources include economic status, religious beliefs, social support, and others. By utilizing these resources, individuals can effectively cope with the pressure and impact of the external environment, leading to a positive and productive high-quality life.

**Figure 1:**
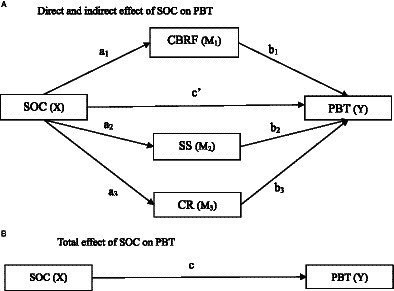
Conceptual diagram of the proposed parallel multiple mediator model. **(A)** Direct and indirect effect of SOC on PBT. **(B)** Total effect of SOC on PBT. PBT, Psychological Birth Trauma; SOC, Sense of Coherence; CBRF, Childbirth-related Fear; SS, Social Support; CR, Childbirth Readiness.

From this perspective, subjective experiences such as CBRF, which are based on past experiences (direct or indirect), can be considered a part of an individual’s internal resources. Childbirth readiness (CR), which is an indicator of an individual’s self-preparation for childbirth, may also be considered an internal resource. Additionally, SS can be viewed as beneficial external resources. We deduced that PBT may be influenced by SOC while CBRF, CR, and SS may play mediate roles in this process. Unfortunately, no published studies have examined these relationships. Guided by the theory of health origins and existing research, we propose the latent pathways of these variables: (1) SOC, CBRF, CR, and SS have impacts on PBT; (2) SOC might exert its effect on PBT through CBRF, CR, and SS. The hypothesized pathways were presented in Figure 1. With the aim of examining the underlying mechanisms of SOC on PBT, the study’s results are essential to triggering future intervention programs that can contribute to preventing PBT.

## 2 Methods

### 2.1 Study design

This cross-sectional study was conducted in obstetric wards of 13 hospitals, comprising 10 comprehensive hospitals and three specialized hospitals, located in mainland China from November 2021 to June 2022. The National Bureau of Statistics had previously divided mainland China into four economic regions, namely the East, Central, West, and Northeast. In accordance with the national principle of regional division, we employed a random selection method to choose seven provinces, municipalities, and autonomous regions from these regions across mainland China. We then selected tertiary and secondary hospitals from these provinces, municipalities, and autonomous regions.

### 2.2 Participants and sampling

Participants were recruited using a convenient sampling method. Inclusion criteria were as follows: (1) ≥20 years old (legal age of marriage for women in China); (2) having given birth within 72 h; (3) exhibiting clear consciousness and good expressive abilities; and (3) providing fully informed consent to participate in the study. Exclusion criteria included premature delivery, stillborn birth, prior diagnosis of psychological disorders such as depression or bipolar disorder, as well as co-concomitant infectious diseases such as AIDS or syphilis.

The sample size estimation in this study was based on the formula for estimating sample size in cross-sectional studies. The literature review indicated that the incidence rate of PBT ranges from 14.3 to 54.4% (Soet et al., 2003; Modarres et al., 2012; Boorman et al., 2014; Bay and Sayiner, 2021; Nagle et al., 2022). The current study adopted the lowest incidence rate of 14.3% to estimate the sample size (Boorman et al., 2014). Using a α of 0.05, and a relative sampling error of 0.15, indicated that 1,049 participants would be needed with a sample power of 0.86. To account for a 20% non-response rate, a total of 1,259 participants were required.

### 2.3 Data collection and measurement

Data were collected through a self-administered online questionnaire by trained nurses in study settings. The questionnaires consisted of five parts:

The first part pertained to **basic information**, including demographic and clinical variables. Demographic variables encompassed age, residence area, occupation status, insurance status, education level, household monthly income *per capita*, marital status, marital satisfaction. Clinical variables included pregnancy sleep status, pregnancy exercise status, pregnancy life events, adverse obstetric history, parity, mode of conception, intention of pregnancy, number of fetuses, abnormal prenatal examination outcomes, fetal development, self-reported pregnancy complications, pressure regarding the fetal gender, mode of delivery, labor analgesia, neonatal weight, self-reported childbirth complications and hospital level.

To evaluate the psychological birth trauma levels of the women, the study employed the **Psychological Birth Trauma Scale (PBTS)**, which is a 15-item self-report scale (Zhang et al., 2023). Each item is rated on a 5-point Likert scale ranging from 1 (not met at all) to 5 (exact match). The total score of the PBTS ranges between 15 and 75, with a higher score indicating more severe psychological birth trauma levels. The PBTS comprises four dimensions: being neglected (four items, Cronbach’s α = 0.878), out of control (four items, Cronbach’s α = 0.804), physiological emotional response (four items, Cronbach’s α = 0.863), and cognitive behavioral response (three items, Cronbach’s α = 0.904). The Cronbach’s α of the total PBTS was 0.937.

**The Sense of Coherence Scale (SOC-3)** was utilized to assess the sense of coherence of the participants (Lundberg and Peck, 1995). The SOC-3 is a three-item scale adapted from Antonovsky’s Sense of Coherence Scale (Antonovsky and Sagy, 1986). The scale comprises three items to represent manageability, meaningfulness, and comprehensibility, respectively. The Agardh scoring method was utilized to calculate the total score (Agardh et al., 2003). Specifically, manageability and meaningfulness were rated on a scale of “often = 3 points, sometimes = 2 points, never = 1 point,” while comprehensibility was scored in reverse, with “often = 1 point, sometimes = 2 points, never = 3 points.” The total score was calculated as the sum of the three dimensions, with higher scores indicating a stronger sense of coherence.

**The Chinese version of the Fear of Childbirth Scale (FOBS)** was employed to estimate childbirth-related fear (CBRF) (Kuipers et al., 2020). The FOBS comprises two items on a visual analog scale, which inquire about the respondent’s feelings toward their approaching delivery. The scale measures the degree of worry and fear on a scale of 0 to 10 for each item, and the total score is computed as the mean of the two items. A higher score on the FOBS indicates a higher level of CBRF. The initial Chinese version of the FOBS exhibited robust internal consistency, with a Cronbach’s alpha of 0.91 (Haines et al., 2011). In the current study, the FOBS yielded a Cronbach’s alpha of 0.89.

**The Chinese version of the Childbirth Readiness Scale (CRS)** was utilized to evaluate childbirth readiness (Mengmei et al., 2022). The CRS is an 18-item questionnaire that comprises four dimensions: self-management, information literacy, birth confidence, and birth plan. Respondents rate each item on a 5-point Likert scale that ranges from 1 (strongly disagree) to 5 (strongly agree). A higher score on the CRS indicates a greater level of childbirth readiness. The CRS has been validated in Chinese pregnant women and exhibits good reliability (Cronbach’s α = 0.94 and split-half reliability = 0.88). In the present study, the CRS reported a Cronbach’s α of 0.96.

**The Chinese version of the Medical Outcomes Study Social Support Survey (MOS-SSS)** was used to evaluate social support (Yu et al., 2004). The MOS-SSS scale comprises one item that evaluates the support network and 19 items that assess the availability of social support in four dimensions: emotional, tangible, affectionate, and positive social interaction. The MOS-SSS is a 5-point Likert scale with the total score ranges from 20 to 100, which a higher score indicating greater social support. The Cronbach’s α for its Chinese version was 0.98 (Boorman et al., 2014). In the present study, the MOS-SSS reported a Cronbach’s α of 0.97.

### 2.4 Data analyses

Data analysis was conducted using IBM SPSS version 27.0. Descriptive statistics were used to present the basic characteristics of the participants. Pearson’s correlation analysis was primarily employed to explore the relationship between the dependent variable and the study variables. A parallel multiple mediator model was analyzed using the PROCESS macro developed by Hayes, which was based on nonparametric percentile bootstrap method, an efficient test method that does not require a population distribution (Preacher and Hayes, 2008; Montoya and Hayes, 2017). As presented in Figure 1, the independent variable X (sense of coherence) had a direct effect (c´) on the dependent variable Y (psychological birth trauma) and indirect effects on Y through three mediators: M1 (childbirth-related fear), M2 (social support), and M3 (childbirth readiness). In Figure 1A, a1 represents the effect of SOC on CBRF, b1 represents the effect of CBRF on PBT, the specific indirect effect of SOC on PBT through CBRF is estimated as a1b1, and so on. The total indirect effect SOC on PBT through three mediators is the sum of the three specific indirect effects. Figure 1B shows the total effect (direct effect + indirect effects) of SOC on PBT, which can be calculated using the formula c = c´ + a1b1 + a2b2 + a3b3. The analysis relied on the estimates of the bootstrap standard errors for hypothesis testing or the bootstrap confidence interval construction in the PROCESS macro to conduct inference for the direct and indirect effects and the contrast of each specific indirect effect. Continuous variables were decentralized before data analysis. Model 4 in Hayes’ PROCESS macro was employed to generate the bias-corrected bootstrap 95% confidence interval (CI) when proceeding with the inference for each effect, with 5,000 bootstrapping samples. An effect was considered statistically significant if the bootstrap 95% CI did not cross zero. All demographic information and clinical characteristics of the patients have been included in the model as covariates to mitigate potential model bias resulting from the loss of variables, and significant covariates were reported alongside the model fitting results.

### 2.5 Ethics approval and consent to participate

All the procedures performed in this study that involved human subjects were in full compliance with the ethical standards of the institutional and national research committee and with the 1964 Helsinki Declaration and its later amendments or comparable ethical standards. Ethical approval was obtained from the Ethics Committee of Tongji Hospital, Tongji Medical College, Huazhong University of Science and Technology (reference number: TJ-IRB20210755). All the participants submitted written informed consent before enrolment in the study. The study is performed in accordance with the relevant guidelines.

## 3 Results

### 3.1 Characteristics of the participants

This study enrolled 1,366 participants totally. In brief, this sample is mainly composed of young first-time mothers (65.45%) under the age of 35 (92.46%), primarily from urban (76.43%) employed (77.96%) individuals. The majority of people have received college or higher education college (72.55%) and have incomes ranging from 3,000–10,000 RMB (61.43%). More detailed information about the basic characteristics of the participants were presented in Table 1.

**Table 1:** Demographic and clinical characteristics of the participants (*n* = 1,366).

Variables	*n* (%)	Variables	*n* (%)
**Age**		**Residence area**	
≤35	1,263 (92.46)	Urban	1,044 (76.43)
>35	103 (7.54)	Rural	322 (23.57)
**Occupation status**		**Insurance status**	
Employed	1,065 (77.96)	With medical insurance	1,138 (83.31)
Unemployed	301 (22.04)	Without medical insurance	228 (16.69)
**Marital status**		**Marital satisfaction**	
Married	1,336 (97.80)	Satisfied	1,323 (96.85)
Others	30 (2.20)	Dissatisfied	43 (3.15)
**Education level**		**Household monthly income *per capita***	
Primary school or lower	22 (1.61)	<3,000¥	306 (22.40)
Junior high school	190 (13.91)	3,000 ~ <5,000¥	407 (29.80)
Senior high school	163 (11.93)	5,000 ~ 10,000¥	432 (31.63)
College or higher	991 (72.55)	≥10,000¥	221 (16.18)
**Pregnancy sleep status**		**Pregnancy exercise status**	
Well	805 (58.93)	Always	174 (12.74)
Moderate	512 (37.48)	Often	507 (37.12)
Poor	49 (3.59)	Occasionally	643 (47.07)
		Never	42 (3.07)
**Pregnancy life events**		**Adverse obstetric history**	
No	1,325 (97.00)	No	1,252 (91.65)
Yes	41 (3.00)	Yes	114 (8.35)
**Parity**		**Mode of conception**	
Nulliparous	894 (65.45)	Conceived naturally	1,302 (95.31)
Multipara	472 (34.55)	Assisted reproduction	64 (4.69)
**Intention of pregnancy**		**Number of fetuses**	
Intended pregnancy	1,056 (77.31)	Singleton pregnancy	1,331 (97.44)
Unintended pregnancy	310 (22.69)	Multiple pregnancy	35 (2.56)
**Abnormal prenatal examination outcomes**		**Fetal development**	
Yes	61 (4.47)	Normal	1,271 (93.05)
No	1,305 (95.53)	Not consistent with gestational age^a^	95 (6.95)
**Self-reported pregnancy complications**		**Pressure regarding the fetal gender**	
Yes	1,205 (88.21)	Yes	214 (15.67)
No	161 (11.79)	No	1,152 (84.33)
**Mode of delivery**		**Labor analgesia**	
Natural birth	1,286 (94.14)	Yes	860 (62.96)
Cesarean section^b^	80 (5.86)	No	506 (37.04)
**Self-reported childbirth complications**		**Neonatal weight**	
Yes	22 (1.61)	Less than 2,500 g	52 (3.81)
No	1,344 (98.39)	2,500 ~ 4,000 g	1,254 (91.80)
		4,000 g or heavier	60 (4.39)
**Hospital level**			
Tertiary general hospital	847 (63.01)		
Tertiary specialized hospital	152 (11.13)		
Secondary general hospital	166 (12.15)		
Secondary specialized hospital	201 (14.17)		

a Smaller than gestational age or larger than gestational age.

b Cesarean section after trial delivery.

### 3.2 Correlations analysis of the independent, dependent and mediate variables

Table 2 displays the results of the correlation analysis conducted on the main study variables. The findings revealed significant correlations (*p* < 0.01) between the dependent variable (PBT) and the independent variables (SOC). Moreover, significant correlations were observed between the mediating variables (CBRF, SS, CR) and both the dependent and independent variables.

**Table 2:** Correlations between the study variables (*n* = 1,366).

	PBT	SOC	CBRF	SS	CR
PBT	1				
SOC	−0.261^**^	1			
CBRF	0.248^**^	−0.098^**^	1		
SS	−0.202^**^	0.202^**^	−0.114^**^	1	
CR	−0.288^**^	0.187^**^	−0.195^**^	0.248^**^	1

**p < 0.01.

PBT, Psychological Birth Trauma; SOC, Sense of Coherence; CBRF, Childbirth-related Fear; SS, Social Support; CR, Childbirth Readiness.

### 3.3 Parallel mediation analysis

**Figure 2:**
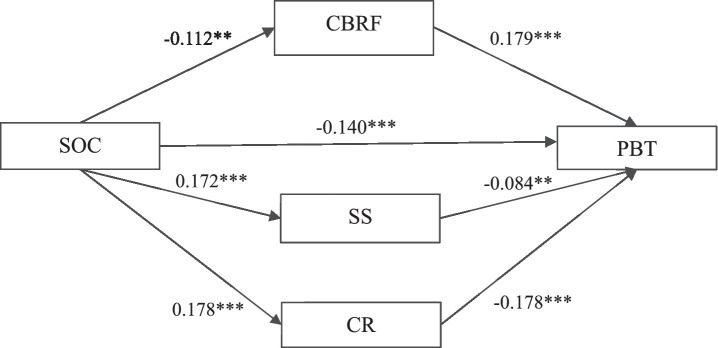
The parallel multiple mediator model of the relationship between Sense of coherence and psychological birth trauma. **p < 0.01; ***p < 0.001. PBT, Psychological Birth Trauma; SOC, Sense of Coherence; CBRF, Childbirth-related Fear; SS, Social Support; CR, Childbirth Readiness.

Figure 2 showed the relationship between SOC, the three mediators and PBT. The total, direct and indirect effects of SOC on PBT, and the associated 95% bootstrap confidence interval were presented in Table 3. The analysis yielded a significant total effect (effect size = −0.206, 95% Boot CI = −0.258 ~ −0.154), direct effect (effect size = −0.140, 95% Boot CI = −0.203 ~ −0.080) and indirect effect (effect size = −0.066, 95% Boot CI = −0.090 ~ −0.044). The indirect effect of SOC on PBT was significantly exerted through CBRF (effect size = −0.020, 95% Boot CI = -0.034 ~ −0.009), SS (effect size = −0.014, 95% Boot CI = −0.028 ~ −0.003) and CR (effect size = −0.032, 95% Boot CI = −0.048 ~ −0.018). No significant difference was found in the contrasts of three specific indirect effect.

**Table 3:** Direct effect and indirect effects of SOC on PBT (*n* = 1,366).

	Effect size	Boot SE	Bootstrap 95% CI		% of total effect
			Boot LLCI	Boot ULCI	
Total effect	−0.206	0.026	−0.258	−0.154	–
Direct effect	−0.140	0.026	−0.203	−0.080	67.98%
Total indirect	−0.066	0.012	−0.090	−0.044	32.02%
Indirect effect through CBRF	−0.020	0.006	−0.034	−0.009	9.75%
Indirect effect through SS	−0.014	0.007	−0.028	−0.003	6.94%
Indirect effect through CR	−0.032	0.008	−0.048	−0.018	15.33%
Contrast 1: CBRF-SS	−0.006	0.009	−0.023	0.012	
Contrast 1: CBRF-CR	0.012	0.010	−0.007	0.031	
Contrast 1: SS-CR	0.017	0.011	−0.004	0.039	

Covariates variables included all demographic and clinical variables. Medical insurance, pregnancy sleep status and adverse obstetric history were found to be significant covariates.

PBT, Psychological Birth Trauma; SOC, Sense of Coherence; CBRF, Childbirth-related Fear; SS, Social Support; CR, Childbirth Readiness.

## 4 Discussion

Guided by the theory of Health Salutogenesis, this study tested the association between SOC, CBRF, SS, CR and PST. While CBRF was positive associated with of PBT, the other three factors (SOC, SS, CR) were negative associated with PBT. Meanwhile, as we proposed, SOC directly exerted its effect on PBT, with CBRF, SS and CR partly mediating its effect on PBT.

We discovered that women who possess a stronger SOC are less likely to experience PBT, with a significant direct effect (effect size = −0.140, % of total effect = 67.98%) in the parallel mediation model. Prior to this study, no research had explored the relationship between SOC and PBT. However, studies focused on post-traumatic stress disorder have reported a correlation between strong SOC and low post-traumatic stress symptoms (Schäfer et al., 2019), indicating a close link between SOC and trauma symptoms. The findings of this study confirmed the protective effect of SOC on PBT. Previous research has established a positive correlation between the extent to which a woman’s childbirth experience deviates from her expectations and a negative childbirth experience (Soet et al., 2003). Furthermore, a perceived lack of control during the childbirth process is a common indicator of a negative experience (Shorey and Wong, 2022). These findings suggest that an individual’s comprehension of the childbirth process and their sense of control over it are crucial determinants of their childbirth experience. The concept of SOC (Antonovsky and Sagy, 1986), which encompasses comprehensibility, manageability, and meaningfulness, can assist individuals in evaluating the challenges of childbirth, mobilizing their internal and external resources to manage stress, and maintaining a belief in the value of nurturing new life. Therefore, SOC may help to alleviate the trauma of childbirth.

Apart from its direct impact on PBT, SOC also indirectly affects childbirth trauma through CBRF, SS, and CR (total indirect effect size = −0.066, % of total effect = 32.02%). Specifically, the present study reveals that CBRF weakens the protective effect of SOC on PBT (specific indirect effect size = −0.020, % of total effect = 9.75%). Extensive literature has demonstrated that both primiparous and multiparous women are at a heightened risk of experiencing traumatic childbirth and subsequently developing PTSD and long-term psychological consequences as a result of CBRF (O’Donovan et al., 2014; Ghanbari-Homayi et al., 2019). The current investigation highlights that, CBRF can not only trigger PBT but also undermine the protective influence of SOC on PBT.

SS and CR were found to enhance the protective effect of SOC on PBT (specific indirect effect size = −0.014, % of total effect = 6.94%; specific indirect effect size = −0.032, % of total effect = 15.33%). SS is an external resource that encompasses general support from partners, family, and friends, as well as professional support from medical personnels. It has been demonstrated to be beneficial in aiding women to cope with various challenges during pregnancy and childbirth, and has been proven to reduce the incidence of traumatic childbirth experiences (Soet et al., 2003; Ghanbari-Homayi et al., 2019). The findings of this study suggest that SS can also work synergistically with SOC to reduce the likelihood of PBT. CR, as a problem-solving skill and a kind of self-efficacy regarding childbirth, is an important internal resource for individuals. If individuals have higher levels of maternal literacy, better self-management during pregnancy, stronger childbirth confidence, and more thorough childbirth plans, they will be better able to cope with the childbirth process (Mengmei et al., 2022). Thus, PBT are less likely to occur in individuals with better CR. Although previous studies have examined the relationship between maternal literacy (an important component of CR) (Soet et al., 2003), confidence in childbirth (Olza et al., 2018) and the childbirth experience, no studies have definitively tested the relationship between CR and PBT. This study extends existing conclusions by demonstrating that CR is a protective factor against PBT and that this protective effect is enhanced when combining SOC. The Health Salutogenesis Theory posits that SOC is advantageous for individuals to effectively mobilize both internal and external resources in order to cope with challenges (Antonovsky, 2022). The findings of this study lend support to this notion. Specifically, if expectant mothers possess a strong sense of SOC, they may be better equipped to recognize and utilize social support and multiple resources to manage the childbirth process. Additionally, they may be more capable of engaging in self-management practices during pregnancy, accumulating knowledge about pregnancy and childbirth, building confidence in their ability to give birth, and making plans for childbirth. These factors, SOC, SS and CR, working together, reduced the likelihood of experiencing PBT.

Regarding the comparison of specific indirect effects, although no significant difference was found in any contracts, the specific indirect effects generated through CR (% of total effect =15.33%) seems to be much higher than those generated through SS (% of total effect = 6.94%) and CBRF (% of total effect = 9.75%). Future studies may yield a significant difference with a larger sample. There are several possible explanations for this phenomenon. CBRF, which is a subjective feeling based on individual’ s past indirect or direct experiences about childbirth (Kuipers et al., 2020), may not produce evident PBT when higher SS and better CR are present. In other words, despite an individual’s fear of giving birth, if she perceives a strong sense of social support and is mentally or physically prepared for the childbirth process, they can effectively respond to the challenges that arise during childbirth, achieve positive outcomes, and avoid the occurrence of PBT. Secondly, compared to SS, which is an external resource, CR is more focused on the individual’s own ability to control the delivery process (Mengmei et al., 2022). As such, it can play a stronger mediating role (although did not significant).

The findings of this study have significant implications for clinical practice. SOC not only directly influences PBT but also exerts an indirect impact through CR, CBRF, and SS, underscoring the necessity of assessing SOC, CR, CBRF, and SS among pregnant women. If pregnant women exhibit weaker SOC, CR, and SS, along with higher CBRF, it suggests a heightened likelihood of future PBT, necessitating increased attention and care from perinatal healthcare professionals. Additionally, based on these assessments, targeted interventions can be designed around these potential intervention targets as part of maternal health promotion initiatives aimed at preventing birth trauma. For instance, cognitive adjustment sessions can be implemented (Kahraman and Gökçe İsbir, 2023; Khademioore et al., 2023) for pregnant women with low SOC and high CBRF alongside routine prenatal care to enhance their sense of comprehensibility, manageability, and meaningfulness regarding childbirth, thereby reducing excessive CBRF and mitigating the risk of PBT. Furthermore, early mobilization of the social support system during pregnancy, coupled with the development of a sufficient childbirth readiness, can empower women to prepare psychologically and physiologically for potential challenges during delivery, fostering confidence and resilience in facing the childbirth in the near future and reducing the likelihood of birth trauma.

The study has limitations. Given its cross-sectional design, the observed relationship between variables cannot be deemed causal. Therefore, there is an urgent need for longitudinal studies to better explain the mechanisms underlying the observed associations. Moreover, the data on CBRF and CR were collected within 72 h after delivery, which raises the possibility of recall bias. Despite these limitations, this study represents a pioneering effort to elucidate the impact of SOC on PBT and its underlying pathways. The findings have significant implications for the prevention and control of PBT. By shedding light on the role of SOC in mitigating PBT, this study provides valuable insights into the potential avenues for intervention and support for mothers who might experience PBT.

## 5 Conclusion

The present study provides compelling evidence that SOC not only exerts a direct effect on PBT but also operates indirectly through CBRF, SS, and CR. Notably, a stronger SOC is associated with a weaker PBT. Furthermore, CBRF attenuate the protective effect of SOC on PBT while SS and CR augment the protective effect of SOC on PBT. These findings underscore the importance of considering SOC, CBRF, SS, and CR as potential targets for interventions aimed at preventing PBT. As such, clinical practitioners and policy makers should pay close attention to these factors in designing effective interventions for preventing PBT. Importantly, interventions that focus on enhancing SOC and CR may hold the greatest promise for preventing PBT.

## Data availability statement

The raw data supporting the conclusions of this article will be made available by the authors, without undue reservation.

## Ethics statement

The studies involving humans were approved by the Ethics Committee of Tongji Hospital, Tongji Medical College, Huazhong University of Science and Technology (reference number: TJ-IRB20210755). The studies were conducted in accordance with the local legislation and institutional requirements. Written informed consent for participation in this study was provided by the participants’ legal guardians/next of kin.

## Author contributions

TZ: Conceptualization, Data curation, Funding acquisition, Project administration, Resources, Supervision, Writing – review & editing. LJ: Formal Analysis, Investigation, Methodology, Software, Validation, Visualization, Writing – original draft. KZ: Conceptualization, Data curation, Writing – review & editing, Methodology. MW: Conceptualization, Data curation, Writing – review & editing. ZZ: Validation, Writing – review & editing. ZH: Validation, Writing – review & editing.
